# Correction: Cao et al. Effective Functional Immunogenicity of a DNA Vaccine Combination Delivered via In Vivo Electroporation Targeting Malaria Infection and Transmission. *Vaccines* 2022, *10*, 1134

**DOI:** 10.3390/vaccines12090994

**Published:** 2024-08-30

**Authors:** Yi Cao, Clifford T. H. Hayashi, Fidel Zavala, Abhai K. Tripathi, Hayk Simonyan, Colin N. Young, Leor C. Clark, Yukari Usuda, Jacob M. Van Parys, Nirbhay Kumar

**Affiliations:** 1 Department of Global Health, Milken Institute School of Public Health, George Washington University, Washington, DC 20052, USA; yicao@gwu.edu (Y.C.); chayashi@gwu.edu (C.T.H.H.); leorcc@gwu.edu (L.C.C.); yukari.usuda@riken.jp (Y.U.); jmvanparys@gwmail.gwu.edu (J.M.V.P.); 2 Department of Molecular Microbiology & Immunology, Johns Hopkins Malaria Research Institute, Bloomberg School of Public Health, Johns Hopkins University, Baltimore, MD 21205, USA; fzavala1@jhu.edu (F.Z.); atripat2@jhu.edu (A.K.T.); 3 Department of Pharmacology and Physiology, School of Medicine and Health Sciences, George Washington University, Washington, DC 20052, USA; hayksimonyan@gwu.edu (H.S.); colinyoung@gwu.edu (C.N.Y.)

The authors would like to make the following corrections to this published paper [1].

During recent verification through DNA sequencing of the DNA plasmid, VR1020-Pfs25, used in this article, the authors identified a minor error in the description of the VR1020-Pfs25 plasmid under Section 2.1 “DNA Vaccine Plasmids”. The statement that “Additionally, all three putative N-linked glycosylation sites in Pfs25 were removed by substitution of asparagine residues with glutamine to prevent N-linked glycosylation [24,25,26,27]” (the second sentence of this subsection) was incorrect. The results of DNA sequencing indicated that these three putative N-linked glycosylation sites in the Pfs25 coding sequence were not mutated. The authors wish to clarify that the presence or absence of these glycosylation sites in Pfs25 does not impact the scientific validity of the results or conclusions in this article. The authors’ previous study (reference 27 cited in the original paper) has demonstrated that the presence or absence of N-linked glycosylation in Pfs25 does not significantly impact the transmission-blocking efficacy of the DNA vaccine VR1020-Pfs25. Therefore, the methods, results, and conclusions drawn in this article remain completely unaltered despite this seemingly minor unintended error.

The sentence should be corrected to “All three putative N-linked glycosylation sites were not mutated in the Pfs25 coding sequence employed [24,25,26,27]”.

The authors state that the scientific conclusions are unaffected. This correction was approved by the Academic Editor. The original publication has also been updated.
