# Supplementary material for: Effective Functional Immunogenicity of a DNA Vaccine Combination Delivered via In Vivo Electroporation Targeting Malaria Infection and Transmission
Source: Vaccines (Basel). 2022 Jul 16;10(7):1134. doi: 10.3390/vaccines10071134 (PMC9323668; doi:10.3390/vaccines10071134)
Supplement: Supplementary file 1 [file vaccines-10-01134-s001.zip › vaccines-1784674-supplementary.pdf]

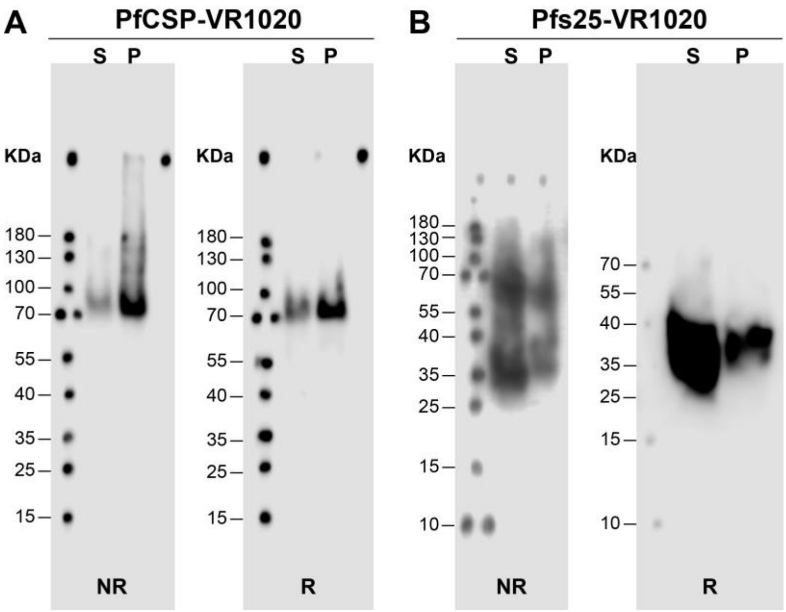

Figure S1. Expression of proteins in mammalian cells. Expi293F cells were transfected with individual vaccine plasmid, PfCSP-VR1020 (**A**) or Pfs25-VR1020 (**B**). Both cell supernatants and cell pellets were tested by Western blotting for protein expression under non-reduced and reduced conditions, using anti-PfCSP monoclonal antibody 2A10 (**A**) and anti-Pfs25 mouse serum (**B**). S, cell supernatant; P, cell pellet; NR, non-reduced condition; R, reduced condition.

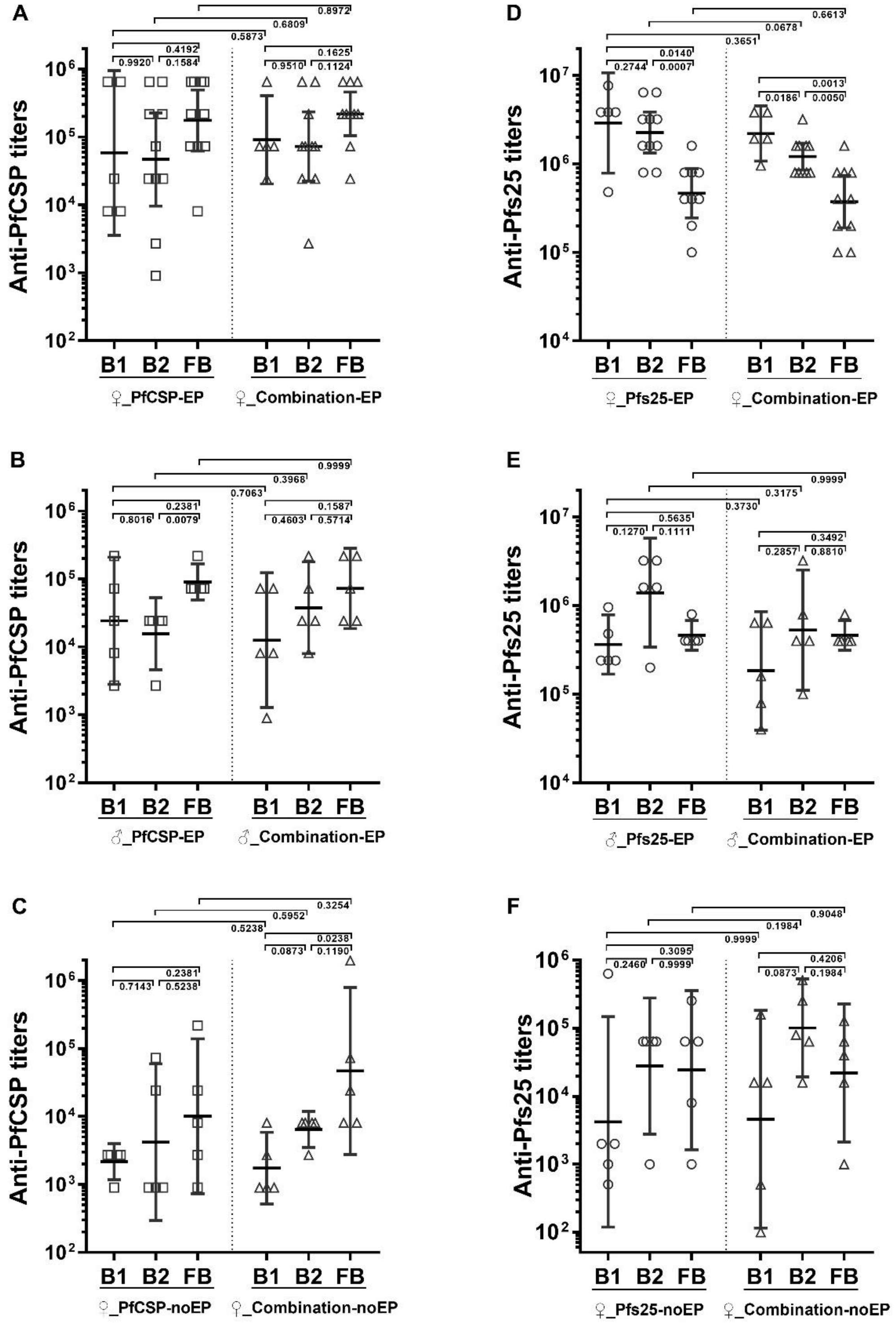

Figure S2. Kinetics of antibody titers in each Balb/c immune group evaluated by ELISA. Pfs25-specific and PfCSP-specific antibodies in Balb/c mouse sera collected at bleed 1 (B1), bleed 2 (B2) and the final bleed (FB) (Figure 1) were assayed and are reported as geometric means (GM) with 95% CI. (**A**–**C**) PfCSP-specific antibody responses in immunized female mice with EP (**A**), male mice with EP (**B**) and female mice without EP (**C**). (**D**–**F**) Pfs25-specific antibody responses in immunized female mice with EP (**D**), male mice with EP (**E**) and female mice without EP (**F**). Statistical analyses were performed using the Mann-Whitney test and *p* values are indicated.

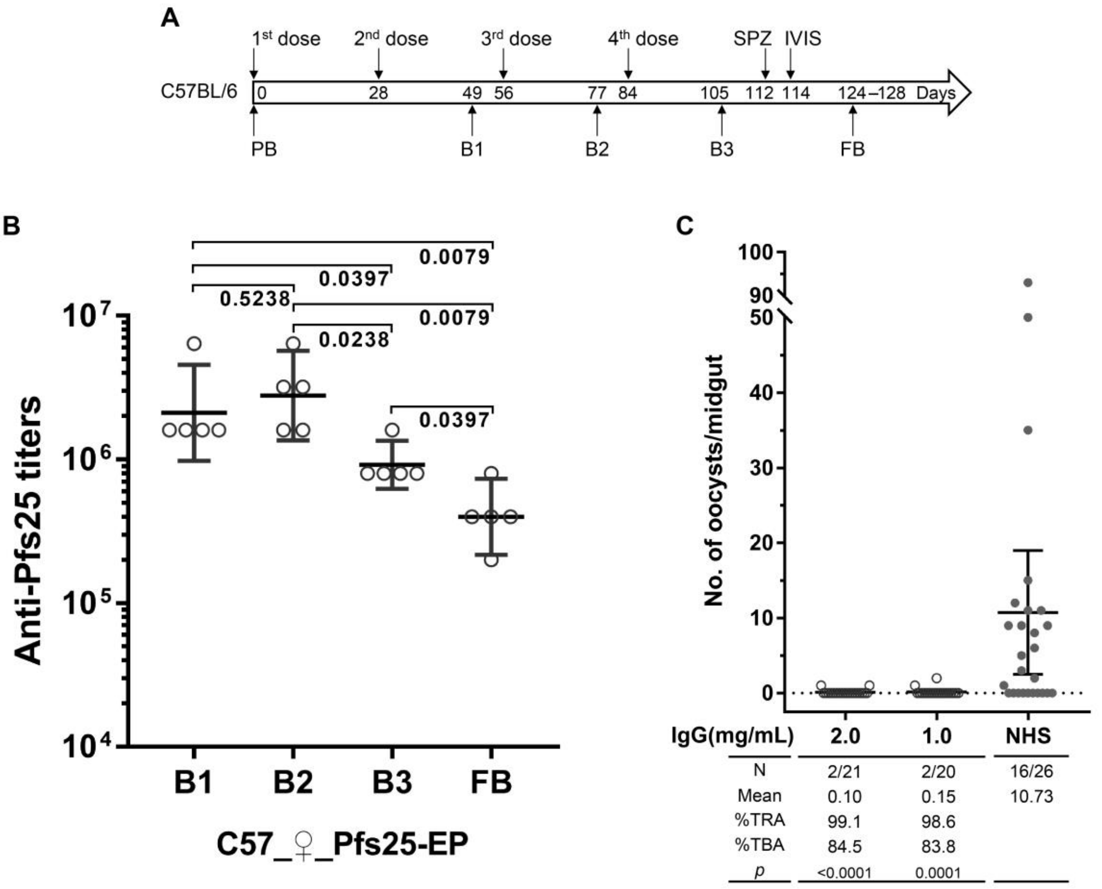

Figure S3. Immunogenicity and transmission reducing activity of Pfs25 DNA vaccine in the C57BL/6 mice. (**A**) Schedule of DNA immunization and sera collection in C57BL/6 mice. A group of female C57BL/6 mice (group 10, n=5) were immunized with 4 doses of Pfs25 DNA vaccine with *in vivo* EP and bled at the indicated time pointes. PB, pre-immune bleed; B1, bleed 1; B2, bleed 2; B3, bleed 3; FB, final bleed. (**B**) Pfs25-specific antibody response in C57BL/6 mice. Antibodies in mouse sera collected in B1, B2, B3 and FB were assayed and are reported as geometric means (GM) with 95% CI. Statistical analyses were performed using the Mann-Whitney test, and *p* values are indicated. (**C**) Transmission reducing activity of Pfs25-specific antibodies of C57BL/6 mice in SMFAs. Total IgGs purified from pooled C57BL/6 mouse sera were assayed in SMFAs at final IgG concentrations of 2 and 1 mg/ml (x-axis), using NHS (no any purified mouse IgG) as the negative control. Each symbol represents the number of oocysts in an individual mosquito, and the bars indicate the arithmetic mean and the 95% CI. Analysis details are summarized in the table beneath panel, as indicated in Figure 4.

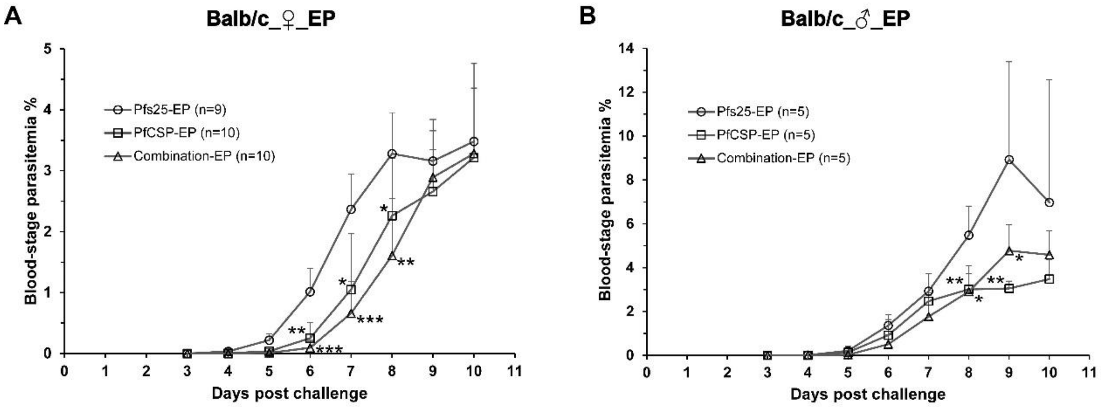

Figure S4. Blood-stage parasitemia in Balb/c mice after challenge with PbPfCSP-GFP/Luc sporozoites. (**A**) Female Balb/c mice in groups 1-3. (**B**) Male Balb/c mice in groups 7-9. Parasitemia of PfCSP-EP and Combination -EP groups were compared to those of the control group (Pfs25-EP) on each day, using the Mann-Whitney test (* $p<0.05$, ** $p<0.01$, *** $p<0.001$).
